# A heterozygous duplication variant of the *HOXD13* gene caused synpolydactyly type 1 with variable expressivity in a Chinese family

**DOI:** 10.1186/s12881-019-0908-6

**Published:** 2019-12-23

**Authors:** Tahir Zaib, Wei Ji, Komal Saleem, Guangchen Nie, Chao Li, Lin Cao, Baijun Xu, Kexian Dong, Hanfei Yu, Xuguang Hao, Yan Xue, Shuhan Si, Xueyuan Jia, Jie Wu, Xuelong Zhang, Rongwei Guan, Guohua Ji, Jing Bai, Feng Chen, Yong Liu, Wenjing Sun, Songbin Fu

**Affiliations:** 1 Laboratory of Medical Genetics, Harbin Medical University, 157 Baojian Road, Nangang District, Harbin, 150081 China; 2 Key Laboratory of Preservation of Human Genetic Resources and Disease Control in China (Harbin Medical University), Ministry of Education, Harbin, China; 3 Department of Hand Surgery the Fifth Hospital of Harbin, 27 Jiankang Road, Xiangfang District, Harbin, 150040 China; 4 Department of Orthopaedic Surgery the Second Affiliated Hospital of Harbin Medical University, Harbin, 150086 China; 5 Department of Hepatopancreatobiliary Surgery the Second Affiliated Hospital of Harbin Medical University, Harbin, 150086 China; 6 Department of Radiology Suihua Cancer Hospital, Suihua, 152000 China

**Keywords:** SPD1, *HOXD13*, Whole genome sequencing, Variable expressivity

## Abstract

**Background:**

Synpolydactyly type 1 (SPD1), also known as syndactyly type II, is an autosomal dominant limb deformity generally results in webbing of 3rd and 4th fingers, duplication of 4th or 5th toes. It is most commonly caused by mutation in *HOXD13* gene. In this study, a five-generation Chinese family affected with SPD1 disease were collected. We tried to identify the pathogenic variations associated with SPD1 involved in the family.

**Methods:**

We used the whole genome sequencing (WGS) to identify the pathogenic variant in this family which was later confirmed by PCR-Sanger sequencing. The genetic variation were evaluated with the frequencies in the 1000 Genome Project and Exome Aggregation Consortium (ExAC) dataset. The significance of variants were assessed using different mutation predictor softwares like Mutation Taster, PROVEAN and SIFT. The classification of variants was assessed according to American College of Medical Genetics and Genomics (ACMG) guidelines.

**Results:**

Our results showed the mutation of 24-base pair duplication (c.183_206dupAGCGGCGGCTGCGGCGGCGGCGGC) in exon one of *HOXD13* in heterozygous form which was predicted to result in eight extra alanine (A) residues in N-terminal domain of HOXD13 protein. The mutation was detected in all affected members of the family.

**Conclusion:**

Based on our mutation analysis of variant c.183_206dupAGCGGCGGCTGCGGCGGCGGCGGC in *HOXD13* and its cosegregation in all affected family members, we found this variant as likely pathogenic to this SPD1 family. Our study highlights variable expressivity of *HOXD13* mutation. Our results also widen the spectrum of *HOXD13* mutation responsible for SPD1.

## Background

Synpolydactyly (SPD) is the combination of two inborn limb deformities i.e. syndactyly and polydactyly, and has an autosomal dominant mode of inheritance. It has been classified into three types SPD1, SPD2 and SPD3 [1]. Generally, the clinical features of SPD1 (MIM#186000) are the webbing of 3rd and 4th fingers, duplication of 4th or 5th toes and some other minor deformities [2, 3].

Limb development involved highly complex process of growth. Transcriptional regulation and signals of many genes are involved in the process (both at molecular and cellular levels) of limb development. Many genes are known to play important role in early limb development e.g. *HOXD13, GLI3, GLI2, SHH, FGF8, WNT7A*, etc. Genetic alteration in any of these genes could lead to limb deformities.

The mutation in *HOXD13* gene located on chromosome 2q31 is responsible for the SPD1 disease. The *HOXD13* gene has two exons that translates into a protein having 335 amino acids and has already been reported several times for SPD1. Different type of mutations at different positions in *HOXD13* leads to SPD1 with severe or less severe phenotypes which includes: i) expansion of polyalanine tract in N-terminal region of HOXD13 [2, 4–6], ii) contraction of the polyalanine tract in N-terminal region of HOXD13 [7], iii) nonsense mutations [8], and iv) missense mutations [9, 10]. Numerous inherited diseases caused due to trinucleotide repeat expansion in specific genes has been reported several times [11–13]. SPD1 is also due to polyalanine expansion in *HOXD13* gene [12].

Here, we report a five-generation Chinese family diagnosed with SPD1. The main objective of this study was to identify the genetic factors causing SPD1 in this family. Since multiple genes reported were related with limb deformities, whole-genome sequencing (WGS) was performed on one of the affected members of the family who showed the typical SPD1 phenotype.

## Methods

### Subjects

**Fig. 1:**
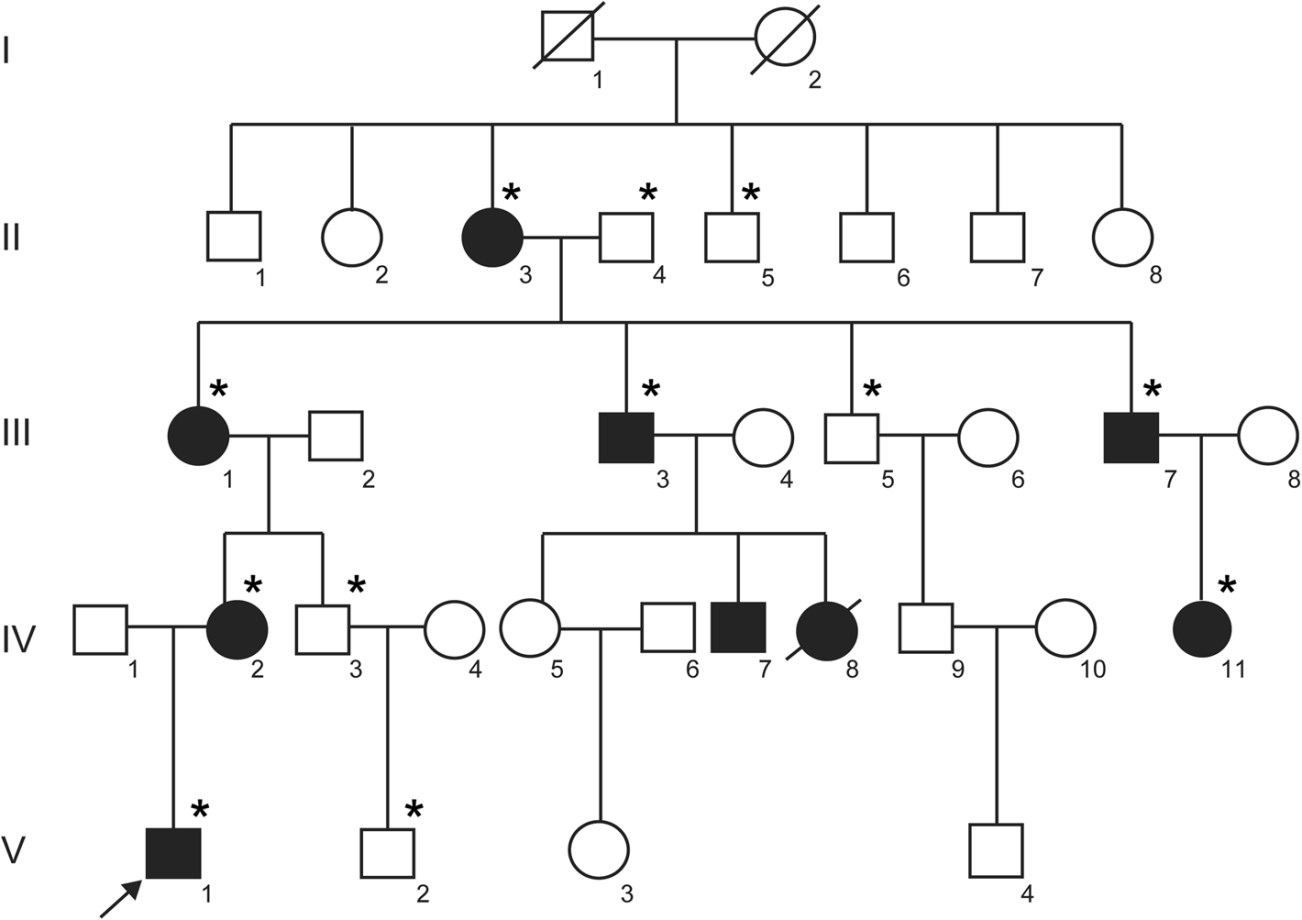
Pedigree of a Chinese family with autosomal-dominant synpolydactyly type 1 (SPD1). Symbols filled with black color are affected members of the family and open symbols represents unaffected members of the family. Arrow indicates the proband (V-1). The sign (*) above the symbols shows members from whom blood samples were attained

In this study, a five-generation pedigree of family with 33 individuals from northern China was studied (Fig. 1). There were nine affected members in the family. We comprehensively examined seven affected members (II-3, III-1, III-3, III-7, IV-2, IV-11, V-1) of the family with limb deformities. The clinical details and blood samples of the two affected persons IV-7 and IV-8 were not available for study. Photographs and radiographs were obtained after detailed clinical examination. Clinical information and peripheral blood were obtained from 12 persons of the family: V-1 (proband), V-2, IV-2, IV-3, IV-11, III-1, III-3, III-5, III-7, II-3, II-4, and II-5. The peripheral blood was collected into a qualified negative pressure vacuum EDTA anticoagulant tube. The study protocol (HMUIRB20180016) was approved by Institutional Research Board of Harbin Medical University and all participants provided signed informed consent.

### Pathogenic gene detection

There are many genes (*SHH*, *GLI3*, *LMBR1*/*ZRS*, *GJA1*, *LRP4*, *BHLHA9*, *APC*, etc.) which have important role in limb development. Genetic alteration in any of these genes could lead to limb deformities. To know about the specific gene mutation that caused SPD1 in the five generation Chinese family, WGS of the blood sample from the typical SPD1 patient IV-2 (affected with both hands and both feet) was performed by Novogene technology limited-liability Company (Beijing, China).

For library generation, 1 μg qualified genomic DNA per sample was needed, which was randomly fragmented to approximately 350-base pair by Covaris S220 sonicator. Then, DNA fragments were end-repaired and ligated to prepare DNA library. After determining the size distribution and concentration, the DNA library was sequenced on Illumina Hiseq X platform for paired-end 150-bp reads. The raw image files were processed by Base Calling analysis. The high-quality sequences are compared to the human reference genome (UCSC: GRCH37/hg19) by Burrows-Wheeler Aligner (BWA) software [14]. The variation information in the sample is detected, counted and annotated. Samtools mpileup and bcftools were used for variant calling to identify SNP, indels while Control-freec is utilized for CNV detection. The variant position, variant type, conservative prediction and other information are obtained at this step through a variety of databases, such as dbSNP, 1000 Genome, the Exome Aggregation Consortium (ExAC) and Human Gene Mutation Database (HGMD). Since we focused in exonic variants, gene transcript annotation databases, as Consensus CDS, RefSeq, Ensembl and UCSC were also applied for annotation to determine amino acid alternation. Using these annotation disease related harmful variations were screened. Finally, literature related to SPD1 was reviewed to identify the potential causative variant.

DNA extraction was performed using the DNeasy Blood & Tissue Kit (Qiagen, #69506, Dusseldorf, Germany) according to the manufacturer’s protocol. Set of primers were designed for exon 1 of *HOXD13* (forward primer GGGAATGGGAGGTGGACCCT, and reverse primer CGTGCGGCGATGACTTGA) using the Primer Premier 5, Software and polymerase chain reaction (PCR) was performed. PCR was performed in a total volume of 20 μl (10 μl 2 × GC buffer, 2 μl 10 × dNTPs, 0.8 μl (10 pmol/μl) forward primer, 0.8 μl (10 pmol/μl) reverse primer, 0.2 μl *taq* DNA polymerase, 4 μl (5 mol/l) Betaine, 2 μl DNA and 0.2 μl water). The PCR reaction started with an initial 5 min denaturation step at 94 °C, followed by 30 cycles of 25 s denaturation (94 °C), 25 s annealing (60 °C), and 60 s extension (72 °C), ending with a final extension step of 5 min at 72 °C. The PCR was carried out on eppendorf Mastercycler nexus GSX1 PCR system. Amplified PCR products were sequenced by TsingKe Biological Technology (Beijing, China) using Sanger sequencing. Results of Sanger sequencing were analyzed using different softwares (Chromas, Megalign and Editseq).

Then the PCR products were purified using QIAquick Gel Extraction Kit following the manufacturer’s protocol. The purified PCR products were ligated to the T linear vector (pEasy-T1 Simple Cloning Kit, #CT111, Transgen, Beijing, China) according to the manufacturer’s protocol and sequenced by TsingKe Biological Technology using Sanger sequencing.

### Bioinformatic analysis of variants

The frequency of variants were evaluated in the 1000 Genome Project (www.1000genomes.org) and ExAC (exac.broadinstitute.org). Suspected variants with frequency < 0.01 were considered for analysis.

The significance of variants were assessed to evaluate their impact on protein structure, protein function and evolutionary conservation using different mutation predictor softwares like Mutation Taster (www.mutationtaster.org), PROVEAN (provean.jcvi.org) and SIFT (sift.bii.a-star.edu.sg). Conservation of the mutation locus was patterned through Aminode (www.aminode.org). The effects of the mutation on protein structure were assessed using SWISS-MODEL (www.swissmodel.expasy.org/).

The standard, guidelines and recommendations for the classification of variants given by American College of Medical Genetics and Genomics (ACMG) were also analyzed for the candidate gene variant [15].

## Results

### Clinical findings

**Fig. 2:**
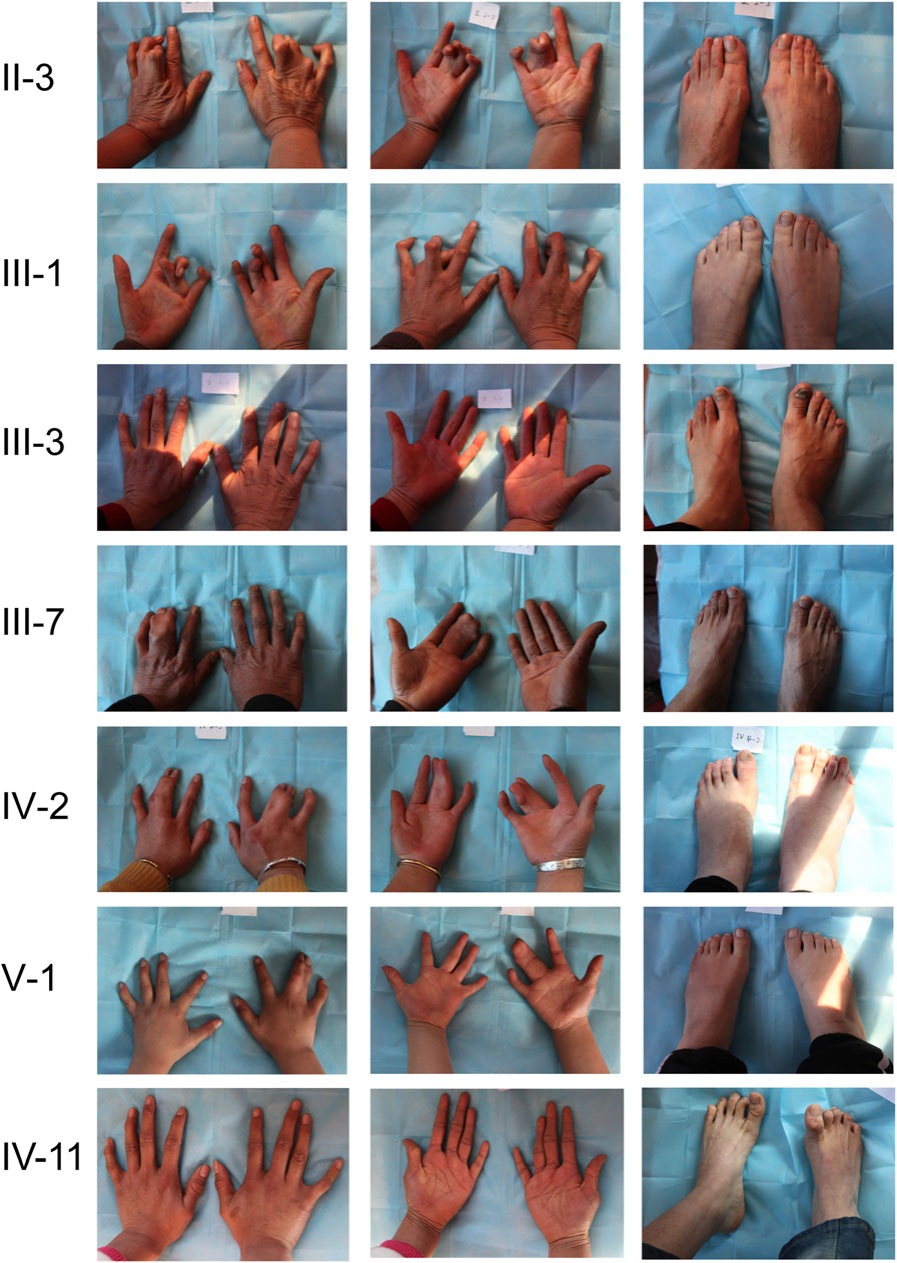
Photographs of the affected members of the family showing hand and foot deformities

**Fig. 3:**
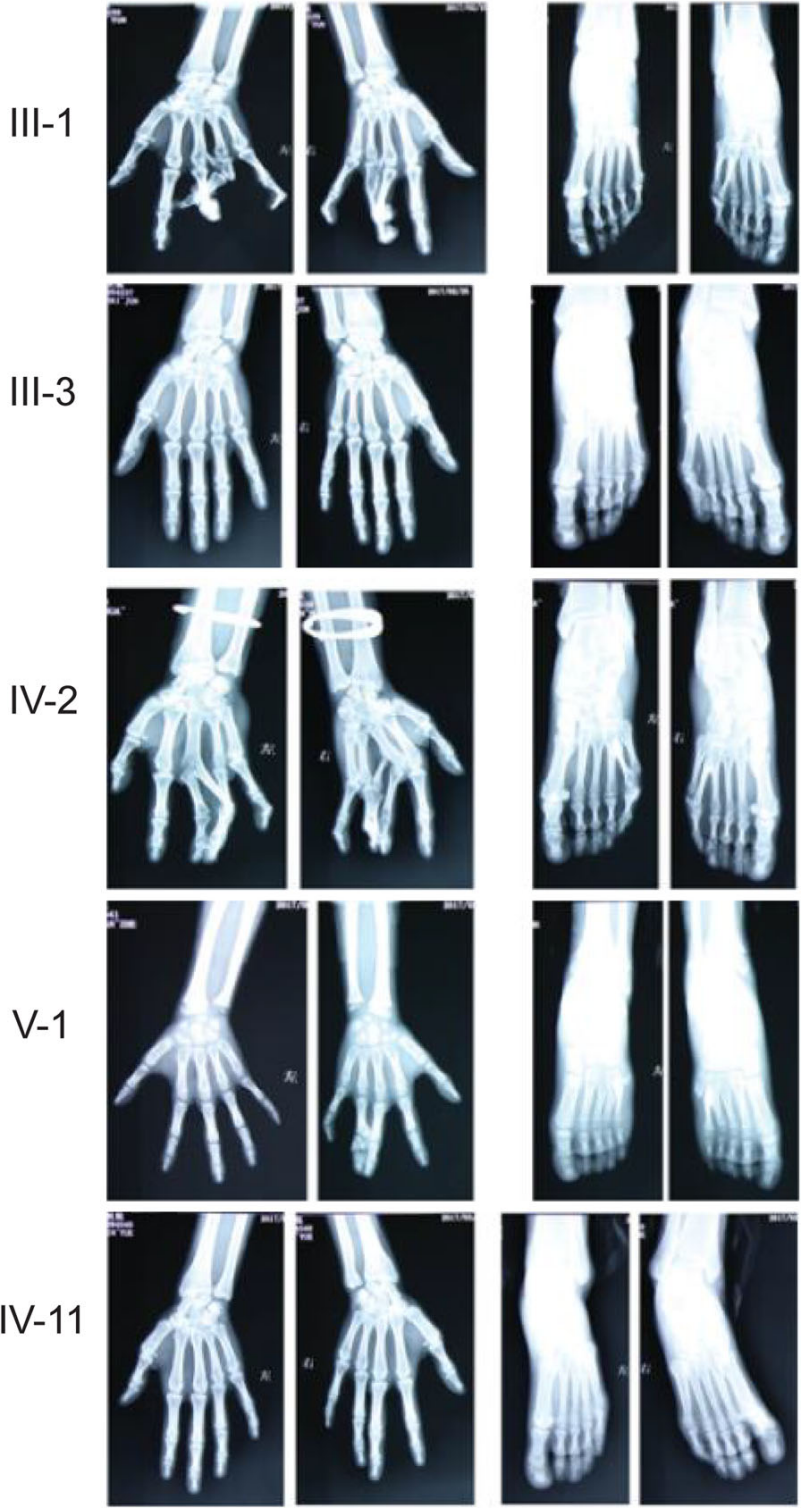
Radiographs of the affected members of the family in which hand and foot deformities are clearly visible

The proband (V-1) was a 12-year-old boy who has been diagnosed with SPD1. The family medical history was further investigated for the disease occurrence. The affected family includes 33 persons in five generations (Fig. 1). Generally, nine members of this family have been suffered from autosomal dominant SPD1. The seven affected members of the family from whom blood samples had been obtained were carefully examined for SPD1 (Table 1). Photographs and radiographs were obtained for affected family members of the family (Figs. 2 and 3). Clinical examination of other family members did not show any sign and symptoms of SPD1 or other limb abnormalities.

**Table 1:** Clinical characteristics of all affected family members

Family ID	Gender	Age (years old)	Deformities in hands	Deformities in feet	Operation
II-3	Female	71	Syndactyly the 3rd and 4th fingers of both hands	None	
III-1	Female	53	Syndactyly the 3rd and 4th fingers of both hands	Little toe polydactyly of right foot	Corrective surgery for both hands, removal of the 6th toes of right foot
III-3	Male	52	None	Little toe polydactyly of right foot	
III-7	Male	45	Syndactyly the 3rd and 4th fingers of left hand	Little toe polydactyly of right foot	
IV-2	Female	31	Syndactyly the 3rd and 4th fingers of both hands	Little toe polydactyly of both feet	Removal of the 6th toe of both feet
IV-11	Female	22	Camptodactyly of the right 5th finger and clinodactyly of the left 5th finger	Camptodactyly of toes and contracture in right hallux	
V-1	Male	12	Syndactyly the 3rd and 4th fingers of right hand	Little toe polydactyly of both feet	Removal of the 6th toe of both feet

### Mutation analysis revealed 24-base pair duplication variant in exon 1 of HOXD13

To identify the disease gene behind SPD1, we performed WGS of patient (IV-2) as she was the only patient with the typical symptoms of SPD1 in the family affected with both hands and both feet. Pathogenic analysis of WGS data revealed 16 pathogenic, 1 likely pathogenic, 2511 variants of uncertain significance (VUS) and 24,319 benign variants. Further analysis of WGS results revealed total of 10,883 missense mutations, 57 frameshift insertions, 183 non-frameshift insertions, and 194 non-frameshift deletions. We have checked all the rare variants falling in syndactyly/limb developmental genes which have been presented in Additional file 1: Table S1. We found their population frequencies were higher than 0.01. The literature related to SPD1 disease was reviewed, and we found that mutation in *HOXD13* gene is the most widely reported for SPD1 disease. Comprehensive analysis of WGS results revealed the duplication of 24-base pair at chr2:176957801–176,957,823 (GRCH37/hg19), which caused a non-frameshift mutation in *HOXD13*. A heterozygous duplication variant c.183_206dupAGCGGCGGCTGCGGCGGCGGCGGC in exon one of *HOXD13* gene was identified in patient (IV-2) by WGS. This mutation is predicted to results in addition of eight extra alanine (A) residues in N-terminal domain of HOXD13 protein. The WGS data can be accessed at SRA accession: PRJNA504318 (www.ncbi.nlm.nih.gov/ sra/PRJNA504318).

**Fig. 4:**
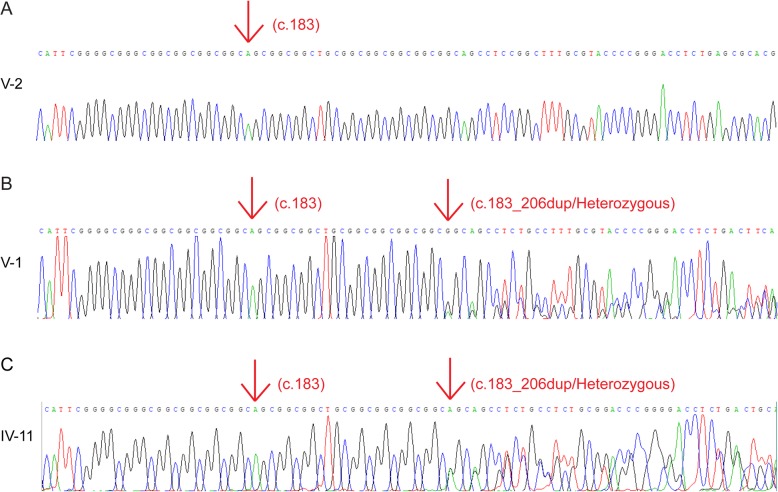
Sequence analysis of *HOXD13* mutation in the Chinese family. **a**, Sanger sequencing result of the unaffected member (V-2) done after PCR showing no double peaks. **b**, Sanger sequencing result of the diseased (V-1) done after PCR showing double peaks at duplication site. **c**, Sanger sequencing result of the affected member (IV-11) done after PCR showing double peaks at duplication site

To determine whether the mutation in *HOXD13* gene was co-segregated in other family members or not, targeted DNA fragments from the patient (IV-2) and other 11 individuals including six patients (II-3, III-1, III-3, III-7, IV-11, V-1) and five normal persons (II-4, II-5, III-5, IV-3, V-2) were first amplified by PCR and then sequenced by Sanger sequencing. The Sanger sequencing results showed that five normal persons did not carry the duplication variant (Fig. 4A. V-2) and all the affected persons carried the 24-base pair duplication variant (Fig. 4B. V-1 & 4C. IV-11).

**Fig. 5:**
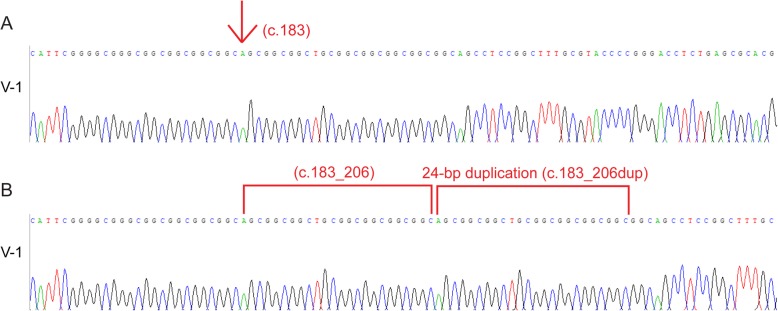
Sequence analysis of *HOXD13* mutation after cloning. **a**, Sanger sequencing result of the proband (V-1) done after cloning showing normal sequence. **b**, Sanger sequencing result of the proband (V-1) done after cloning showing 24-base pair of duplication

Further investigation was performed into the two affected persons (V-1 and IV-2) sequence by ligating the purified PCR products with the T linear vector, then followed by subcloning and Sanger sequencing. The sequencing results showed that both patients (V-1 and IV-2) carried one normal sequence (Fig. 5A. V-1) and one mutated sequence (Fig. 5B. V-1) which indicated that mutation exists in heterozygous form. The normal sequence and the mutation sequence can be accessed at GenBank: MK290763 for normal one, and MK290762 for the *HOXD13* c.183_206dupAGCGGCGGCTGCGGCGGCGGCGGC.

### Bioinformatic analysis of the identified 24-base pair duplication variant of HOXD13

#### Frequency

The 24-base pair duplication variant in exon one of *HOXD13* was neither found in 1000 Genome project nor in ExAC and has been absent in these population data bases.

#### In silico predictions

Mutation taster predicted polymorphism for it, while PROVEAN predicted it as deleterious with the score of − 2.719. SIFT predicted the variant as neutral.

#### Evolutionarily constrained region of HOXD13

**Fig. 6:**
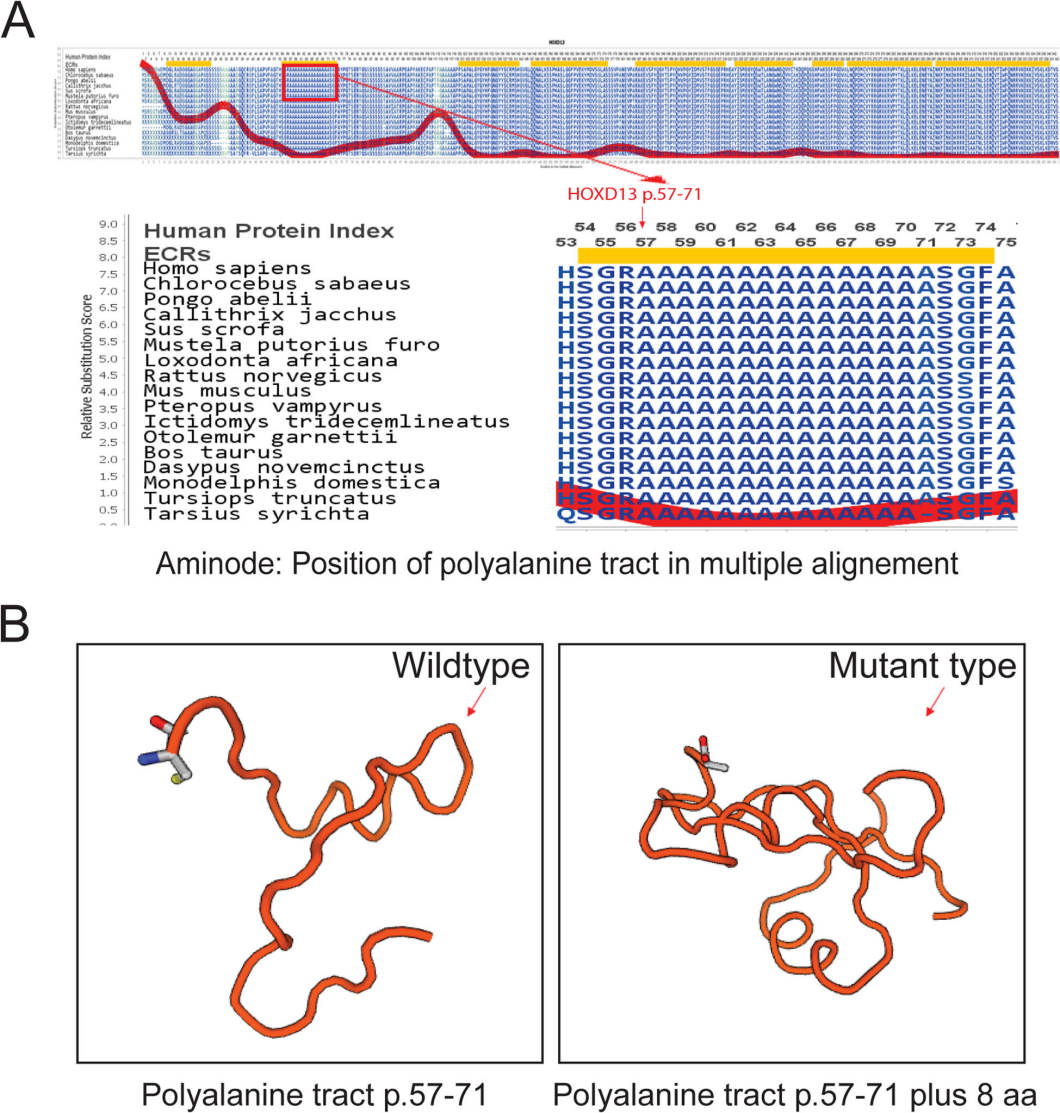
Bioinformatic analysis of the HOXD13 duplication variation (c.183_206dup). **a**, Conservation analysis of HOXD13 using Aminode showed that polyalanine tract (p.57–71) of the HOXD13 protein is conserved among different species. **b**, Protein structure of HOXD13 wild type (p.57–71) and mutant type (p.57–71 plus 8 aa) showed that duplication variation locally affected the shape and size of HOXD13 protein, the images were created using SWISS-MODEL (www.swissmodel.expasy.org/)

Evolutionarily constrained regions (ECRs) of *HOXD13* according to Aminode revealed that the polyalanine tract (p.57–71) of the *HOXD13* protein was conserved among different species, such as *Mus musculus* and *Rattus norvegicus*, etc. (Fig. 6A).

#### Homology model for the HOXD13 protein

A homology model of wildtype and mutant *HOXD13* proteins revealed that the 24-base pair duplication variant encoding eight extra alanine (A) residues in the polyalanine tract locally affected the shape and results in increase in size of the protein (Fig. 6B).

### ACMG evaluation for 24-base pair duplication variant of HOXD13

According to classification of ACMG for assessing the pathogenicity of different variants, the 24-base pair duplication variant of *HOXD13* is “likely pathogenic” as it is in mutation hot spot (PM1), absent in population data bases (PM2), co-segregated in affected members of the family (PP1), computational evidence showed deleterious effect (PP3), and highly specific disease phenotype (SPD1) with single gene (*HOXD13*) (PP4). Thus, according to ACMG the 24-base pair duplication mutation of *HOXD13* has two moderate (PM1, PM2) and more than two supportive (PP1, PP3, PP4) evidences of pathogenicity, fulfilled the criteria of ACMG for “likely pathogenic” variant.

## Discussion

Synpolydactyly is a limb deformity in which hands and feet of the patient get affected and inherited in an autosomal dominant pattern from one generation to the next. Mutation in *HOXD13* gene located in chromosome 2q31 is responsible for the SPD1 disease. In this study, we found a heterozygous duplication variant (c.183_206dupAGCGGCGGCTGCGGCGGCGGCGGC) in exon 1 of *HOXD13* [NM_000523.3] in a five-generation Chinese family with SPD1.

Complete cosegregation of the variant with the disease was evident in this family, which is the most reliable way to evaluate the pathogenicity of a variant. In our case, duplication of 24-base pair in exon 1 of *HOXD13* were found in all affected members (II-3, III-1, III-3, III-7, IV-2, IV-11 and V-1) of the family. It encodes additional eight alanine (A) residues in the polyalanine tract at N-terminal domain of HOXD13 protein. There are 15 alanine residues present in N-terminal domain of wildtype HOXD13 protein, due to 24-base pair duplication mutation consequently the number of alanine residues increases to 23 (15 aa plus 8 aa).

Some previous studies also reported addition of 7–14 extra polyalanine tract expansions in families with SPD [2, 4–6, 16–22], but our results are different from the previously reported families in terms of variable expressivity. Goodman et al. reported the families from British and German populations with SPD having duplication of 24-base pair in exon one of *HOXD13* and eight extra polyalanine expansions [5], while Xin et al. reported a family with SPD in Chinese population having eight extra polyalanine expansions in HOXD13 [2]. But the exact site of the duplication was not mentioned in both studies. Chinese family reported by Xin et al. showed SPD features that were consistent with our SPD family as most of the SPD patients had fusion of fingers 3rd and 4th and little toe polydactyly of right or left foot, but our patients have more severe features of SPD. In our SPD family, three out of seven SPD patients have both hands affected with syndactyly, while in case of family reported by Xin et al. only two out of seven SPD patients had syndactyly in both hands. Furthermore, according to Xin et al. older generations were more severely affected than younger generations, while in our SPD family older and younger generations were equally affected i.e. family members II-3, III-1, III-7, IV-2 and V-1 showed severe complications of SPD1.

Several other different types of mutations had also been reported for SPD1 in *HOXD13*, i.e. deletions [7, 23], nonsense mutations [8, 24, 25] and missense mutations [9, 10, 26–32], but most common amongst all was expansion of polyalanine repeats [2]. Therefore, polyalanine repeats in N-terminal region of HOXD13 is a mutational hot spot and mutation in this region will comes under category PM1 (moderate evidence of pathogenicity) according to classification of ACMG for classifying pathogenic variants [15].

*HOXD13* encodes highly conserved DNA binding transcription factors that helps other genes to initiate their transcription. Hyperexpansion of DNA-triplet repeats as a result of 24-base pair duplication in exon 1 of *HOXD13* may lead to altered transcription or translational activities which can result in defective mRNA and ultimately defective proteins. Polyalanine expansions in other transcription factors had been reported to be linked with human birth defects such as deformities of digits and other structures [6].

In vertebrates *HOX* genes have significant role of morphogenesis which translates into a group of some greatly conserved transcription factors [33]. Deep investigation of five *HOX* genes (*HOXD9-HOXD13*) in mouse and chick revealed that it has extremely vital role in development of limbs [34]. These genes seem to play the same role in humans as mutations in *HOXD13* gene were previously reported to be responsible for SPD1 cases [2, 4, 5, 7].

In case of variable expressivity or gene expression, some family members show severe complications of synpolydactyly (II-3, III-1, III-7, IV-2, V-1) while other did not show the same complications (III-3, IV-11) (Fig. 2). The five generation Chinese family showed from minor to more severe limb deformities, which are beneficial for further understanding of SPD1 clinical features in future. Camptodactyly and clinodactyly of 5th finger and toes and contracture in right foot’s hallux have been added to the list of possible phenotypes caused by HOXD13 polyalanine expansion mutations*.*

The variable phenotypes of SPD1 patients in this family predicts that some other factors (environmental or genetic) can contribute to the expression of *HOXD13*, as some genetic factors (e.g. gene modifiers) and epigenetic factors also found to play an important role in controlling the expression of any specific gene. Phenotypic heterogeneity of any disorder is because of interaction of responsive gene with other associated genetic factors or modifier genes. *Raj* et al. also showed that mutation in that locus or gene which is critical for developmental process could lead to variable expressivity [35]. Though, number of mutations in *HOXD13* has been reported up till now to be linked with SPD1 disease, but still a clear genotype-phenotype association is not well established. Detail investigation of 32 SPD1 families by Malik et al. also revealed a weak genotype-phenotype correlation [1].

## Conclusions

We successfully identified duplication mutation (c.183_206dupAGCGGCGGCTGCGGCGGCGGCGGC) in exon 1 of *HOXD13* [NM_000523.3]. Based on clinical data, cosegregation analysis, in silico predictions and ACMG assessment, we classified the *HOXD13* 24-base pair duplication variant as likely pathogenic and the main cause of SPD1 in this family. Our results widen the genotypic spectrum of *HOXD13* mutations that are responsible for SPD1. The phenomena of variable expressivity were quite obvious in this family. In comparison with previous studies it is established that the variable expressivity is the common phenomena in SPD1. More research is required in the area to find out the genetic factors behind phenotypic heterogeneity.

## Supplementary information

**Additional file 1: Table S1.** Mutations in limb development genes found in WGS

## Data Availability

The WGS data can be accessed at SRA accession: PRJNA504318 (www.ncbi.nlm.nih.gov/ sra/PRJNA504318).

## Abbreviations

ACMG: American College of Medical Genetics and Genomics
BWA: Burrows-Wheeler Aligner
ECRs: Evolutionarily constrained regions
ExAC: Exome Aggregation Consortium
HGMD: Human Gene Mutation Database
PCR: polymerase chain reaction
SPD1: Synpolydactyly type 1
VUS: Variants of uncertain significance
WGS: Whole Genome Sequencing
