# Supplementary material for: A heterozygous duplication variant of the HOXD13 gene caused synpolydactyly type 1 with variable expressivity in a Chinese family
Source: BMC Med Genet. 2019 Dec 23;20:203. doi: 10.1186/s12881-019-0908-6 (PMC6929446; doi:10.1186/s12881-019-0908-6)
Supplement: Supplementary file 1 — Additional file 1: Table S1. Mutations in limb development genes found in WGS [file 12881_2019_908_MOESM1_ESM.docx]

Table 1 Mutations in limb development genes found in WGS data

| Gene | Sequence | cDNA change | AA change | Mutation type | 1000 Genome frequency | ExAC frequency |
| --- | --- | --- | --- | --- | --- | --- |
| *GLI2* | NM_005270 | c.3466G>T | p.A1156S | missense SNV | 0.508986 | 0.6296 |
| *GLI2* | NM_005270 | c.3916G>A | p.D1306N | missense SNV | 0.567093 | 0.6464 |
| *GLI2* | NM_005270 | c.801G>A | p.S267S | synonymous SNV | 0.827276 | 0.9401 |
| *GLI2* | NM_005270 | c.3939 A>G | p.P1313P | synonymous SNV | 0.51238 | 0.6344 |
| *GLI3* | NM_000168 | c.2993C>T | p.P998L | missense SNV | 0.421526 | 0.4558 |
| *GLI3* | NM_000168 | c.547A>G | p.T183A | missense SNV | 0.603634 | 0.563 |
| *BHLHA9* | NM_001164405 | c.237A>G | p.L79L | synonymous SNV | 0.374601 | 0.2647 |
| *LRP4* | NM_002334 | c.4660 A>G | p.S1554G | missense SNV | 0.367612 | 0.4942 |
| *LRP4* | NM_002334 | c.3256A>G | p.I1086V | missense SNV | 0.445887 | 0.5849 |
| *APC* | NM_000038 | c.5465T>A | p.V1822D | missense SNV | 0.865415 | 0.7981 |
